# Large medial meniscus extrusion and varus are poor prognostic factors of arthroscopic partial meniscectomy for degenerative medial meniscus lesions

**DOI:** 10.1186/s13018-022-03045-0

**Published:** 2022-03-18

**Authors:** Tao Xu, Liuhai Xu, Xinzhi Li, You Zhou

**Affiliations:** Department of Orthopedics, Affiliated Renhe Hospital of China Three Gorges University, Yichang, 443001 Hubei China

**Keywords:** Degenerative medial meniscus lesions, Arthroscopic partial meniscectomy, Prognostic, Influencing factor

## Abstract

**Background:**

The indications and efficacy after arthroscopic partial meniscectomy (APM) for degenerative medial meniscus lesions (DMMLs) have been controversial. The purpose of this study was to identify predictors of unfavorable clinical and radiologic outcomes after APM for DMMLs and to choose appropriate indications and improve treatment efficacy.

**Methods:**

A total of 86 patients with DMMLs undergoing APM were retrospectively reviewed. The mean follow-up time was 32.1 months. Clinical outcomes (including Lysholm score) and radiographic results (including Kellgren-Lawrence grade (K–L grade: 0/1/2/3/4) were evaluated at preoperative and final follow-up. Preoperative prognostic factors, including gender, age, Body Mass Index (BMI), Hip–Knee–Ankle (HKA), Medial Posterior Tibial Slope (MPTS), Medial Meniscus Extrusion (MME), K–L grade, occupational kneeling, and cartilaginous condition (Outerbridge grade ≤ 2, VS ≥ 3), for relatively unfavorable (fair or poor grade) Lysholm and progression of K–L grade, were investigated by multivariate logistic regression analysis. Receiver operating characteristic curve was used to identify a cutoff point for the extent of medial meniscal extrusion that was associated with the final Lysholm score.

**Results:**

A significantly improved postoperative Lysholm score (84.5 ± 9.7) compared with the preoperative score (63.8 ± 9.3) (*P* < 0.001), but a progression of K–L grade (20/36/30/0/0–15/27/25/19/0) (*P* < 0.001). The adverse prognostic factor of Lysholm score was the advancing age (OR 1.109, *P* = 0.05) and HKA (OR 0.255, *P* < 0.001). The adverse prognostic factor of K–L grade progression was MME (OR 10.327, *P* < 0.001). The cutoff point for the relative value of preoperative medial meniscal extrusion associated with relatively unfavorable Lysholm scores was 2.05 mm (Area = 0.8668, *P* value < 0.0001, Sensitivity = 62.16%, Specificity = 93.88%).

**Conclusions:**

Clinically, varus alignment, large MME, and older age were found to predict a poor prognosis after APM for DMMLs. The preoperative extent of MME can be used as a predictive factor for osteoarthritis in APM. Patients with varus and MME should avoid APM. High tibial osteotomy may be an effective treatment strategy.

## Introduction

The DMMLs had a high incidence in the middle old-age. About 25% were aged 50–59, 35% aged 60–69, and 45% aged 70–79. The 2016 European Society for Sports Traumatology, Knee Surgery and Arthroscopy (ESSKA) Meniscus Consensus considered that nonsurgical treatment was the primary choice for DMMLs [1]. However, not all DMMLs respond to conservative treatment. For patients with normal or near-normal X-rays but abnormal MRI, surgical treatment was recommended after the failure of nonsurgical treatment for 3 months, or those who are undergoing persistent pain, and/or presenting mechanical symptoms (e.g., compression, locking and clicking) [2].

In past decades, a partial meniscectomy was considered a “traditional” treatment and was used widely, with more than 500,000 procedures performed annually in the USA. Between 1997 and 2017, 1.1 million APM were carried out in the United Kingdom [3, 4]. Although a partial meniscectomy can provide symptomatic relief in short-term follow-up, it can lead to progression of degenerative arthritis in mid- to long-term follow-up [5, 6]. A large number of relevant studies have shown that APM does not provide any additional benefit for DMMLs compared with non-operative or sham surgical treatment, and does not solve the underlying biomechanical abnormalities, which may increase the risk of osteoarthritis [7–10]. The determination of preoperative prognostic factors was crucial to improve the outcome of surgery and delay the progress of osteoarthritis. Investigators have noted that before the surgical procedure for DMMLs, the role of varus alignment in clinical outcomes and the entire progress of osteoarthritis should be considered [11, 12]. Seil et al. [2] believed that the surgical decision-making process must include the negative imaging factors such as knee osteoarthrosis (K–L ≥ grade 2), age, terminal chondropathies, lateral meniscectomy and lower limb malalignment, for patients with persistent pain and no mechanical symptoms. However, the preoperative factors that affect the prognosis of DMMLs with APM are rarely used in quantitative studies, and previous studies mainly used reviews to describe their influencing factors.

Thus, the purpose of this study was to identify predictors of unfavorable clinical and radiologic outcomes after APM for DMMLs. We hypothesized that malalignment, poor cartilage status, medial meniscus extrusion may result in unsatisfactory clinical and radiographic outcomes in follow-up examinations for DMMLs.

## Methods

### Study population

This study protocol has been approved by the Institutional Review Board. We retrospectively reviewed 426 patients who underwent arthroscopic meniscus surgery between January 2018 and January 2020. All cases were retrospectively analyzed to verify the demography and clinical characteristics of the enrolled patients.

**Fig. 1:**
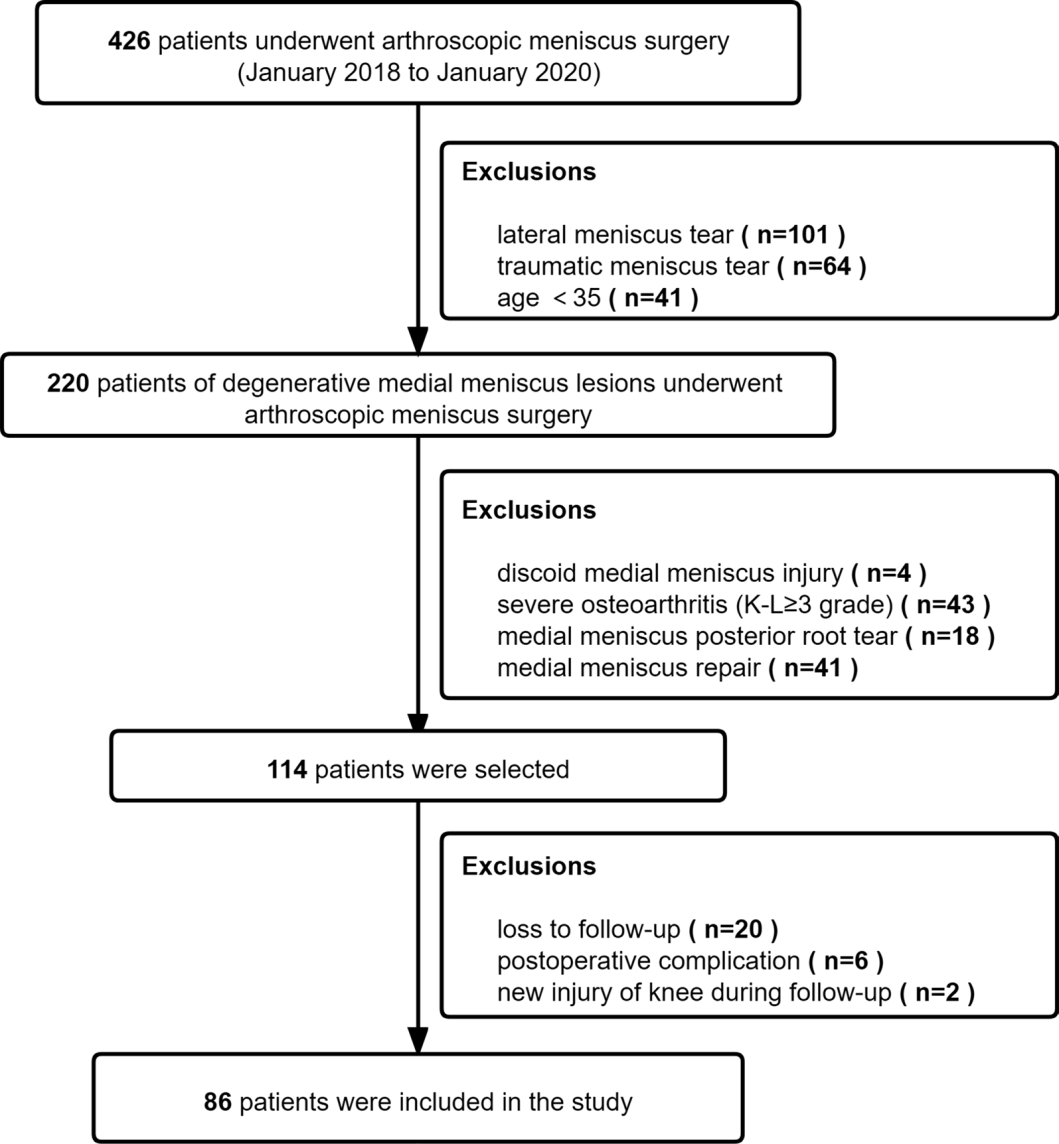
Flowchart of patient inclusion in the study

**Fig. 2:**
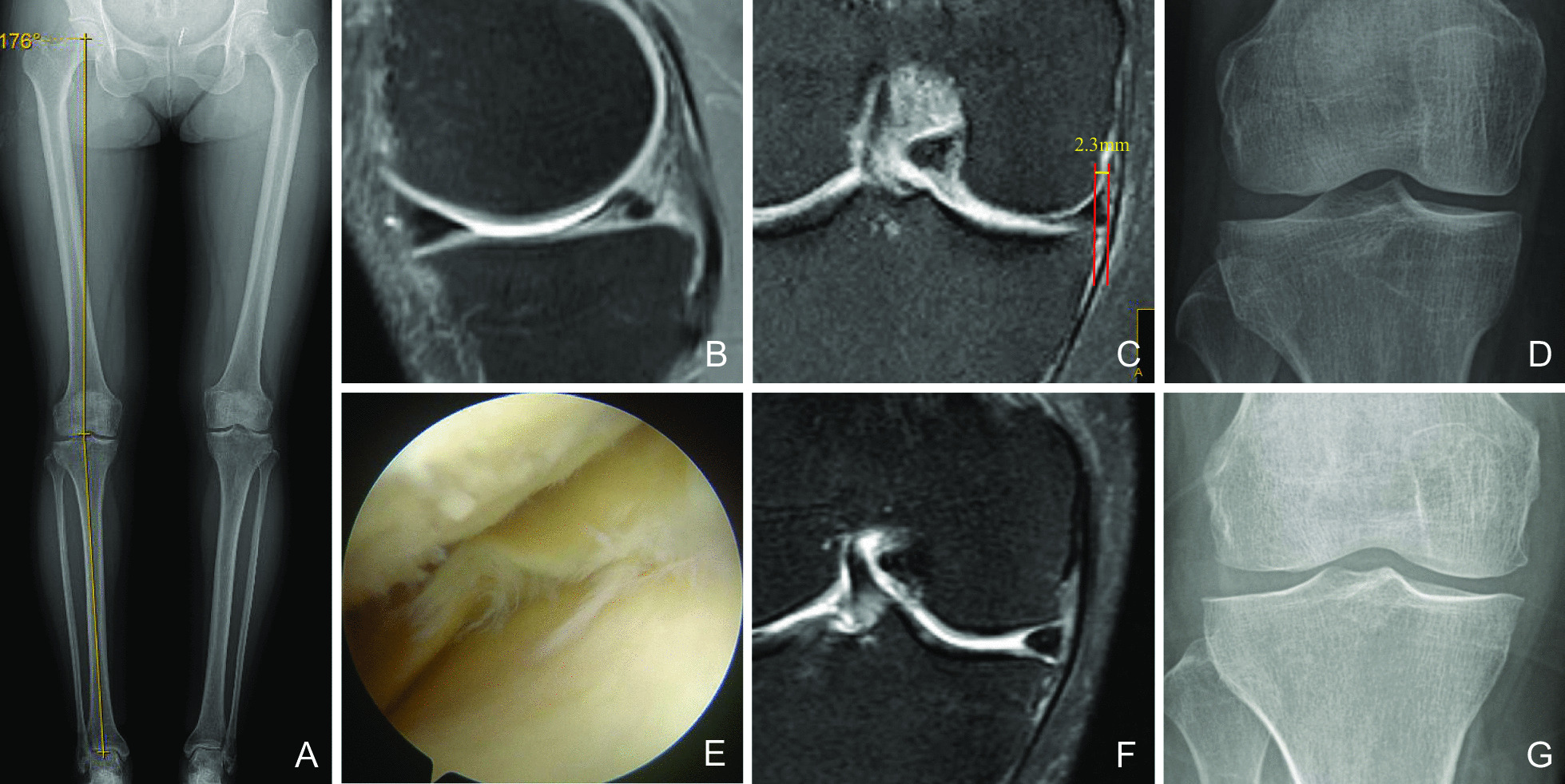
A woman, 51 years old, the posterior horn of DMMLs of the right knee. HKA:176° (**A**), the posterior horn of DMMLs and MME 2.3 mm (**B**–**C**), K–L grades 0 preoperatively (**D**), the DMMLs of posterior horn under arthroscopic and Outerbridge classification 2 degree (**E**), wedge-shaped posterior edge of medial meniscus at 6 months postoperatively (**F**), K–L grades level 1 at 38 months postoperatively (**G**)

Patients were included in the current study according to the following criteria: (1) No any history of significant acute trauma in a patient older than 35 years. (2) A degenerative meniscus lesion was usually characterized by linear intrameniscus MRI signal (including a component with horizontal pattern) communicating with the inferior meniscus surface on at least two image slices, and lesion was the body and (or) posterior horn of the medial meniscus (Fig. 1). (3) Arthroscopic knee surgery confirmed DMMLs. (4) No or mild degenerative osteoarthritis (K–L ≤ 2 grade). (5) Patients who failed after strict non-operative treatment for 3 months, or persistent mechanical pain and symptoms (e.g., compression, locking and clicking) (Fig. 2). Exclusion criteria: (1) lateral meniscus tear, (2) traumatic meniscus tears, (3) degenerative lesions occurring in young patients, especially in athletes (4) discoid medial meniscus injury, (5) severe osteoarthritis (K–L ≥ 3 grade), (6) medial meniscus posterior root tear, and (7) medial meniscus repair. Twenty patients who satisfied the inclusion criteria of the current study were not included because of loss to follow-up. Two patients were excluded due to new injury of the knee during follow-up. Six patients who had a postoperative complication were excluded due to iatrogenic chondral injury caused by tight medial compartment. No patient included in the study underwent knee arthroplasty for osteoarthritis. Thus, 86 patients were included (Fig. 1).

All surgical procedures were performed by a single senior sports medicine doctor. No additional procedures, such as microfractures or chondroplasty, were performed. These lesions generally did not qualify for suture repair because of severe degeneration in the meniscus margin, instability, poor blood supply, and low healing rate, such as small vertical flap or complex tear.

We recommended lifestyle modifications to avoid deepflexion of the knee to all patients. The second day after surgery, increase the strength of the muscles (i.e., ankle pump exercise, quadriceps isometric contraction, and straight leg raise. Step by step, 3–4 groups a day, each group is about 15 min, the knee can be iced for 20 min after exercise to prevent swelling and soreness). Partial weight-bearing exercises were carried out at 1 week postoperatively. Full weight-bearing strengthening exercises were allowed at 1 week postoperatively. Jogging was allowed at 1 month postoperatively.

### Clinical and imaging results

Lysholm score was evaluated at preoperative and final follow-up. Clinical outcomes were evaluated by a physician who was not involved in the procedure. Preoperative results were compared with the final results to assess whether APM could maintain significantly improved outcomes clinically.

**Fig. 3:**
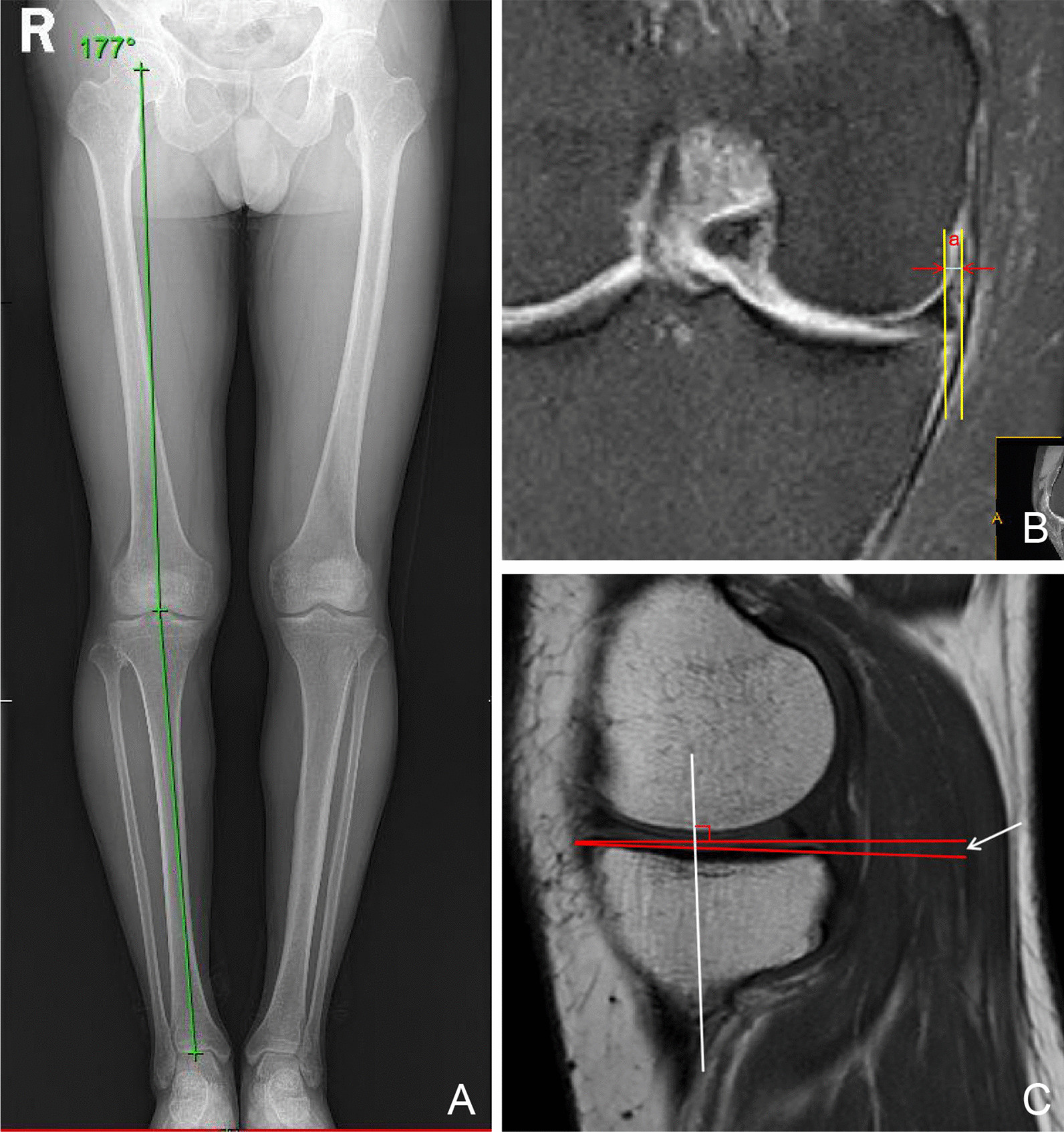
**A** HKA; **B** Measurement of medial meniscus extrusion. Two vertical lines were drawn perpendicular to the articular horizontal line at the outer edges of meniscus and the outer edge of the articular cartilage of the tibial plateau. Absolute extrusion was defined as the distance (a) between the outer edge of the articular cartilage and the outer edge of the medial meniscus; **C** The included angle formed by the connection between the vertical line of the longitudinal axis of the tibia and the highest point of the anterior and posterior edge of the widest sagittal plane of the medial tibial plateau is the MPTS

K–L grades (0/1/2/3/4) (frontal and lateral weight-bearing radiographs) were assessed and compared at preoperative and final follow-up. The maximal extrusion distances of the meniscus were measured as the distance between the external margin of the tibial plateau area and that of the tibial meniscus surface [13]. HKA was the angle formed by the connection between the center of the femoral head, the center of the knee joint and the center of the ankle joint. For varus, HKA < 180°, and for valgus, HKA > 180°. We used the method described by Hudek to determine the medial plateau posterior tibial slope using the proximal tibial anatomic axis and a tangent to the uppermost anterior and posterior edges of the medial plateau [14]. Intraoperative arthroscopy confirmed the cartilaginous status of the medial compartment, which was graded according to the Outerbridge classification (Fig. 3).

### Affected prognostic factors and statistical analysis

SPSS24.0 software was used for statistical analysis. Spearman correlation coefficient analysis and U test were utilized to analyze the factors affecting postoperative prognosis. Spearman correlation coefficient analysis included correlations between age, BMI, HKA, MPTS, MME, and final clinical outcomes. The impact of categorical variables (including gender, cartilage status, occupational kneeling, and K–L progression) on the final clinical outcomes was assessed in the U test analysis.

In order to determine preoperative imaging factors that might lead to a relatively adverse prognosis, multivariate logistic regression analysis was performed for patients with fair or poor Lysholm grade and progression of K–L grade, and the OR of preoperative prognostic factors was calculated by stepwise regression method. "Unfavorable" clinical outcomes were defined as "fair" or "poor" Lysholm score. Lysholm score were divided into four grades: 91–100 scores for excellent, 84–90 scores for good, 65–83 scores for fair, and scores ≤ 64 for poor. The "unfavorable" radiographic outcome was considered progression of K–L grade. Logistic regression analysis was used to assess the preoperative data to determine the risk factors affecting prognosis.

## Results

Preoperative demographic data, preoperative and final results are shown in Table 1. 6 patients who had iatrogenic chondral injury were excluded due to tight medial compartment. Two patients had infrapatellar fat pad injury due to obscuration of the vision. And the pain has been eliminated by rehabilitation and physical therapy 2 weeks after the operation. Three patients still had medial joint line pain. It has shown a significantly improved postoperative Lysholm score (84.5 ± 9.7) compared with the preoperative score (63.8 ± 9.3) (*P* < 0.001), but a progression of K–L grade (20/36/30/0/0 ~ 15/27/25/19/0) (*P* < 0.001) in the image. Thirty-eight of 86 patients (44.2%) showed K–L grade progression. Nonetheless, there was no K–L grade progression to grade 4, and no total knee arthroplasty conversion patients (Table 1).

**Table 1:** Baseline characteristics of the participants allocated to APM (*n* = 86)

*Variable*	
Sex, males (*N*)/females (*N*)	41/45
Age, y	53.2 ± 7.0
BMI [$\overline{x} \pm s$]	23.7 ± 2.4
Follow-up period (mo)	32.1 ± 7.8
HKA (°)	176.8 ± 1.8
MPTS (°)	6.4 ± 2.2
MME (mm)	1.6 ± 0.9
Cartilage status, 0/1/2/3/4 (Outerbridge classification)	20/23/27/16/0
Occupational kneeling, yes/no	8/78

	Preoperation	Postoperation	*P* value
Lysholm score	63.8 ± 9.3	84.5 ± 9.7	0.000*
Kellgren–Lawrence grade, 0/1/2/3/4	20/36/30/0/0	15/27/25/19/0	0.000^&^

Continuous variables are shown as mean ± SD

*APM* arthroscopic partial meniscectomy, *BMI* body mass index, *HKA* hip–knee–ankle, *MPTS* medial posterior tibial slope, *MME* medial meniscal extrusion, *K–L grade* Kellgren–Lawrence grade

^*^u-test

^&^Fisher exact test

In Spearman correlation analysis, advancing age, smaller HKA and larger MME were negatively correlated with the final Lysholm score (Table 2). In the U test, Outerbridge grade ≥ 3 and K–L grade progression were associated with unfavorable final Lysholm score (Table 3). In multiple logistic regression analysis, advancing age (OR 1.109; *P* = 0.05) and smaller HKA (OR 0.255; *P* < 0.001) were an important factor causing unfavorable Lysholm score. Larger MME (OR 10.327; *P* < 0.001) is an adverse factor leading to the progression of K–L grading (Table 4).

**Table 2:** Results of correlation of clinical factors by Spearman correlation analysis

Continuous variables*	Coefficient	*P*
Preoperative factors		
Age, y	− 0.586	0.000
BMI	0.013	0.906
HKA, °	0.822	0.000
MPTS, °	− 0.211	0.051
MME, mm	− 0.794	0.000

*BMI* body mass index, *HKA* hip–knee–ankle, *MPTS* medial posterior tibial slope, *MME* medial meniscal extrusion

*Coefficients of correlation between continuous variables and clinical results were investigated by spearman correlation coefficient

**Table 3:** Results of categorized clinical factors by *U* analysis

Nominal variables	Final Lysholm score	*P*
Sex, *n*		
Male, 41	82.9 ± 10.3	0.188
Female,45	86.0 ± 9.0	
Cartilage status (Outerbridge classification)		
≤ 2, 70	87.6 ± 7.4	0.000
≥ 3, 16	71.1 ± 6.8	
occupational kneeling, *n*		
Yes, 8	81.5 ± 8.3	0.206
No, 78	84.8 ± 9.8	
K–L grade progression, *n*		
No progression, 48	89.4 ± 7.1	0.000
Progression, 38	78.3 ± 9.1	

*u*-test

*K–L grade* Kellgren–Lawrence grade

**Table 4:** Multivariate logistic regression analysis

Dependent variables	Significant variables	OR	*P* value	95% CI
Lysholm, fair or poor^&^, *n* = 37	Age, y	1.109	0.050	1.102–1.232
	HKA, °	0.255	0.000	0.131–0.499
Progression of K–L grade, *n* = 38	MME	10.327	0.000	4.009–26.602

*CI* confidence interval, *HKA* hip–knee–ankle, *K–L* grade Kellgren–Lawrence grade, *OR* odds ratio

^&^The Lysholm score is categorized into the following 4 grades: excellent = 91–100, good = 84–90, fair = 65–83, and poor < 64

*Significant variables (*P* value ≤ .05) are descripted from age, HKA, cartilage status grade ≥ 3 and medial meniscal extrusion using forward stepwise method to evaluate preoperative prognostic factors leading to unfavorable outcomes

**Fig. 4:**
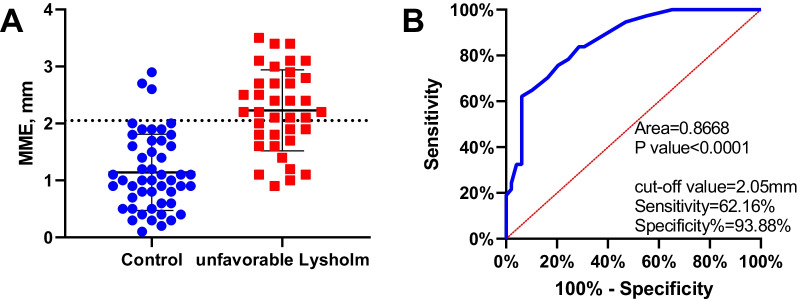
**A** Scatter plot of MME of DMMLs; **B** ROC curve of independent risk factors of the final Lysholm score in DMMLs

The cutoff point for the relative value of preoperative medial meniscal extrusion associated with relatively unfavorable Lysholm scores was 2.05 mm (Area = 0.8668, P value < 0.0001, Sensitivity = 62.16%, Specificity% = 93.88%) (Fig. 4).

## Discussion

The principal findings of this study were that advancing age and smaller HKA were the primary reasons of poor postoperative clinical outcomes. Larger preoperative MME was a risk factor of postoperative K–L grading progression. Therefore, patients should be overall evaluated at the preoperative period before APM. Meanwhile, a better treatment strategy should be generated to maximize the knee function performance of patients and delay the progression of osteoarthritis.

The purpose of APM was to excise the irreparable meniscus margin of tear and retain the peripheric stability hoop so as to relieve symptoms caused by the meniscus lesions, compression and locking. The treatment of DMMLs by APM has attracted more and more attention from researchers but is still controversial. A multicenter, randomized, controlled trial showed that APM was not significantly superior to sham surgery in patients with DMMLs without knee osteoarthritis [15]. Currently, a large number of literature have reported that APM was not supported to be applied in first-line treatment of patients with DMMLs [16–18]. Sihvonen et al. [18] performed APM on 70 patients with DMMLs and found that Lysholm score showed statistically significant difference in improvement compared with the preoperative condition, but APM did not improve knee symptoms or function better than the arthroscopic surgery group and showed no statistically significant difference. Furthermore, APM was associated with a slightly increased risk of knee osteoarthritis within 5 years after surgery. Therefore, for patients with conservative treatment and second-line APM treatment, the factors of poor prognosis should be analyzed, and a more reasonable treatment plan should be developed to improve the prognosis of patients.

The results of this study showed that lower limb malalignment was the main influencing factor for poor prognosis in clinical outcomes of DMMLs patients undergoing APM. Willinger et al. [19] found that the lower limb alignment and the amount of medial meniscus resection had significant effects on tibiofemoral contact pressure through researching the biomechanics of eight fresh cadavers. MME with varus increased mean contact pressure and peak contact pressure in the medial compartment, while lateral displacement of the line prevented overloading in the medial compartment. Willinger et al. [20], in another fresh cadaveric study, showed that the common layer tearing of DMMLs and intact meniscus have similar contact area and peak contact pressure. When APM was applied to one or two layers of the horizontal tearing, the contact area was significantly reduced and peak contact pressure was significantly increased. Both APM and varus resulted in increasing peak contact pressure in the medial compartment. The degenerative meniscus still had a protective effect on subchondral bone, even in osteoarthritis [21]. Therefore, the partial meniscus resection should be avoided in clinical practice to maintain the original biomechanical behavior. If partial meniscus resection was necessary, the structure of the meniscus should be retained as much as possible. In addition, the lower limb alignment played a key role in the load distribution between the medial and lateral of knee compartments, and varus was a potential risk factor for medial osteoarthritis. Therefore, clinicians should avoid APM in patients with layer tearing of DMMLs and considered alternatives (e.g., high tibial osteotomy in patients with varus) to reduce excessive stress in the medial compartment and removed the risk factors caused by varus to relieve knee symptoms and delay the further development of osteoarthritis [11]. More and more literature supported that DMMLs may be an early signal of knee osteoarthritis rather than a clinical problem of meniscus intervention alone [12]. For example, one study showed that there was no significant association between meniscus lesions and frequent knee pain in the middle-aged and the older, the occurrence of associated osteoarthritis should be taken into account [22]. The varus was an independent risk factor for medial osteoarthritis [23]. For patients with symptomatic DMMLs combined with varus, the osteotomy for line correction could be conducted as a preventative early measure to completely remove the risk factors without waiting for the confirmation of radiated osteoarthritis [12]. In this regard, we also conducted a study on the relationship between the lower limb alignment and DMMLs. We confirmed that varus was a risk factor of DMMLs and also one of the influencing factors for the poor prognosis of patients with DMMLs after APM.

In addition, MME was a risk factor of K–L grade progression in DMMLs patients after APM. The cutoff point for the relative value of preoperative MME associated with relatively unfavorable Lysholm scores was 2.05 mm. Kim et al. [24] had similar results with this study, which showed that the degree of meniscus degeneration and the manner of meniscus lesions (including horizontal, flap fissure and compound tear) were related to the MME, which had attracted more and more attention in clinical meniscus dynamic function evaluation and meniscus lesions diagnosis [25]. Meniscus extrusion was considered to be an indirect sign of meniscal pathology. It may be accompanied by loss of meniscus function, followed by deformity and loss of articular cartilage in the medial compartment, and eventually developed into osteoarthritis. There was a closed relationship between lower limb alignment and MME. Willinger et al. [25], through researching the biomechanics of eight fresh cadaveric knee specimens, investigated the relationship between MME and joint contact stress when the lower limb alignment was poor. The varus resulted in a higher MME and a corresponding increase in mean contact pressure and peak contact pressure compared to neutral or valgus. Goto [26], through retrospective multiple logistic regression analysis, showed that the varus was significantly correlated with MME, and the greater varus, the greater MME, and the more severe the osteoarthritis. Meniscus extrusion suggested dysfunction of meniscus, which was one of the causes of spontaneous osteonecrosis and was related to the development of spontaneous osteonecrosis [27]. The increased stress of femoral condyle of MME and varus may lead to enlargement of spontaneous osteonecrosis and changes of the secondary osteoarthritis [28]. Preoperative MME was negatively correlated with the clinical outcomes of partial meniscectomy, which could be used as a predictive factor of osteoarthritis after partial meniscectomy. The larger MME increased the risk of knee osteoarthritis. If APM was performed, this could increase the progression of osteoarthritis [15, 29].

Achtnich et al. [30] showed that the increase in age, BMI and load was significantly and positively correlated with the increase in meniscus extrusion. Varus, MME, DMMLs, and knee osteoarthritis were closely related and mutually influenced [1].The patients with lower limb malalignment and MME should be comprehensively evaluated. Patients accompanied with varus could choose osteotomy to remove the risk factors of osteoarthritis caused by lower limb malalignment, to reduce the pressure of medial compartment, and to relieve the pain at medial compartment. Astur et al. [31] studied the relationship between MME width and high tibial osteotomy, suggested that open wedge high tibial osteotomy could reduce MME and improve clinical efficacy and reactivate function. In addition, patients with a postoperative MME less than 1.5 mm had better clinical outcomes and activity levels than those with a postoperative MME of 1.5 mm or greater. Jing et al. [32] showed that for patients with medial meniscus posterior root tear, after high tibial osteotomy combined with root repair, the degenerated medial femoral condyle cartilage regenerated during the second-look arthroscopic findings. Therefore, for patients with DMMLs, a more satisfactory prognosis can be achieved by identifying the adverse factors affecting the operation, a comprehensive preoperative evaluation, a strict indication control and a reasonable treatment method.

The study had several limitations. First, this was a non-randomized retrospective study, so there could be a selective bias. Second, due to the absence of second-look arthroscopic findings, there was a lack of more intuitive evaluation indicators. Finally, because DMMLs were a signal of early osteoarthritis, the follow-up time was short, and there was a lack of long-term follow-up datum of imaging and clinical results.

## Conclusions

In conclusion, advancing age, varus, and increased MME were risk factors leading to poor postoperative prognosis of APM for DMMLs and to progression of osteoarthritis. Lower limb alignment and MME should be assessed in each case of medial meniscal lesions. The most appropriate treatment plan could be selected, and corresponding intervention could be given in the early stage of osteoarthritis to improve treatment outcome, so as to effectively delay the progression of osteoarthritis.

## Data Availability

Please contact author for data requests.

## Abbreviations

APM: Arthroscopic partial meniscectomy
DMMLs: Degenerative medial meniscus lesions
K–L grade: Kellgren–Lawrence grade
BMI: Body mass index
HKA: Hip–knee–ankle
MPTS: Medial posterior tibial slope
MME: Medial meniscus extrusion
OR: Odds ratio
